# Targeting Menin in Acute Myeloid Leukemia: Therapeutic Advances and Future Directions

**DOI:** 10.3390/cancers16223743

**Published:** 2024-11-06

**Authors:** Sandhya Dhiman, Vikram Dhillon, Suresh Kumar Balasubramanian

**Affiliations:** 1 Department of Oncology, Karmanos Cancer Center, School of Medicine, Wayne State University, Detroit, MI 48201, USA; dhimans@karmanos.org (S.D.); vdhillon@med.wayne.edu (V.D.); 2 Department of Oncology, Neal Cancer Center, Houston Methodist Hospital, Houston, TX 77030, USA

**Keywords:** menin, KMT2A, MLL, NPM1, epigenetics, fusion proteins, acute myeloid leukemia (AML), targeted therapy

## Abstract

Germline mutations in the *MEN1* gene encoding menin protein cause multiple endocrine neoplasia type 1 (MEN1) syndrome. Recent evidence suggests that inhibiting the interaction of menin with its crucial oncogenic protein partners represents a promising therapeutic strategy to AML. Menin plays a critical role in lysine methyltransferase 2A (*KMT2A*)-gene-rearranged and *NPM1*-m acute leukemias, both associated with adverse outcomes with current standard therapies, especially in the relapsed/refractory setting. Disrupting the menin–KMT2A interaction affects the proleukemogenic *HOX/MEIS* transcription program. This disruption leads to the differentiation of *KMT2A*r and *NPM1*-m AML cells. Small molecular inhibitors of the menin–KMT2A interaction target the central cavity of MEN1 to inhibit the MEN1-KMT2A interaction and could target a similar transcriptional dependency in other leukemia subsets, broadening their therapeutic potential. These agents, both as monotherapies and in combination with synergistic drugs, are undergoing preclinical and clinical evaluation with promising early results. With the growing literature around menin inhibitors in AML, we discussed the biology of menin, its mechanism of action, its interacting partners in leukemia, possible inhibitors, their implications, synergistic drugs, and future therapeutic strategies in this review.

## Simple Summary

Menin, encoded by the *MEN1* gene, scaffolds many interacting proteins through which it regulates important gene expression critical for cellular growth and differentiation. Menin is also critical in regulating genes that drive cancer growth in acute myeloid leukemia (AML), and more recently, targeting menin’s interaction with *KMT2A* has emerged as a promising therapeutic strategy for treating AML, especially with *KMT2A* rearrangement and *NPM1* mutations. The menin inhibitors currently in development demonstrate promising early efficacy and may offer a more targeted approach than traditional chemotherapy and possibly better outcomes. This review will discuss the evolution of menin as a target in AML therapeutics, the clinical development of various menin inhibitor and outcome data from the latest clinical trials, and the understanding of its resistance mechanisms and strategies to improve outcomes and future directions.

## 1. Introduction

### 1.1. Menin as a Target in Leukemia

Decoding the genetic drivers in leukemogenesis has led to new target discoveries in recent years. Cytotoxic chemotherapy had been used in treating AML for decades until molecularly directed agents were discovered. However, none has translated into a cure yet. Among the various novel mechanisms currently being evaluated in clinical trials, inhibition of menin’s interaction with its protein partners has recently gained interest in the treatment of AML.

Menin is a tumor suppressor scaffold protein encoded by the *MEN1* gene, and germline mutations are linked to multiple endocrine neoplasia 1 (MEN1) syndrome [1]. Menin also serves as a crucial oncogenic cofactor for the *MLL* (mixed lineage leukemia) or *KMT2A* (lysine methyltransferase 2A) gene, and the fusion of MLL with one of its fusion partners results in chimeric MLL fusion proteins that block hematopoietic differentiation, resulting in leukemogenesis [2].

The crystal structure of menin reveals four major domains: an N-terminal domain, two middle domains referred to as the thumb and palm domains, and a C-terminal finger domain [3]. A prominent feature within the thumb and palm domains is a large internal cavity that contains several tetratricopeptide repeat (TPR) motifs [4]. The central cavity, enclosed by helical subdomains, retains a conserved architecture even upon binding to protein ligands. This structural feature functions as the primary binding site for protein–protein interactions of MLL and its fusion partners, such as JunD [5].

**Figure 1:**
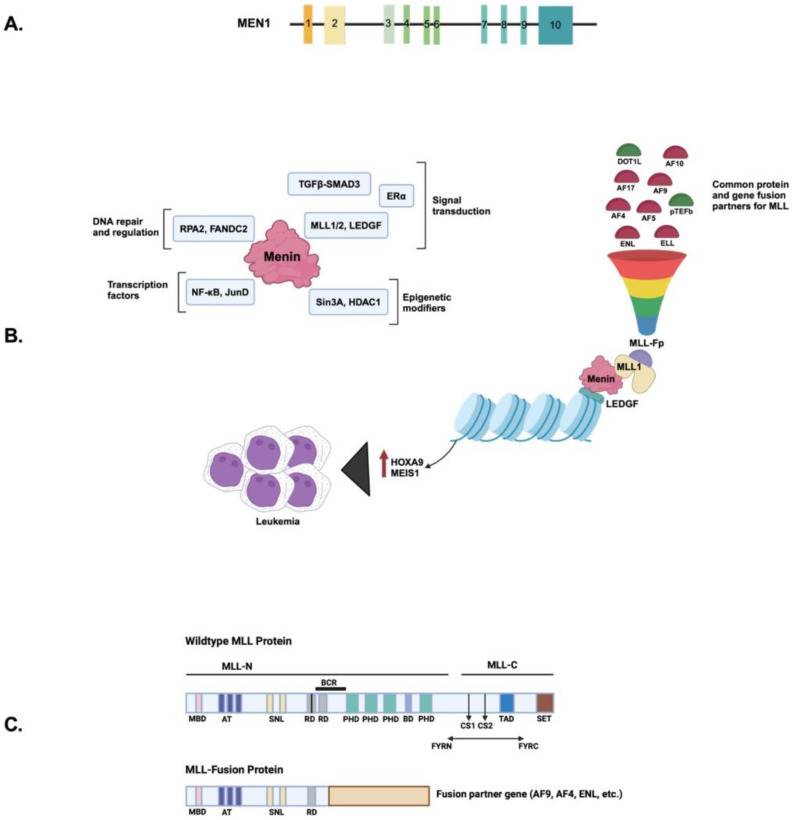
Structure of menin and its functions: (**A**) Chromosomal location of *MEN1* on chromosome 11q13 with ten exons. (**B**) The scaffolding function of menin regulating gene expression by interacting with different classes of chromatin regulators and transcription factors (protein fusion partners in red; gene fusion partners in green; MLL-Fp is MLL fusion partners represented above the funnel). (**C**) Structural representation of wild-type MLL with the different functional domains indicated by colors. MBD is the menin-binding domain; AT is the AT hooks; SNL are the Speckled Nuclear Localization domains; RDs are the repression domains; the black line in the first RD is the CXXC domain; BCR is the breakpoint cluster region; MLL fusion proteins are the result of chromosomal rearrangements between N-terminal MLL up to the BCR and any of the 80 fusion partners; PHDs are the four PHD fingers; BD is the bromodomain; CS1 and CS2 are the taspase-1 cleavage sites; FYRN to FYRC is the region where MLL-N and MLL-C will interact after cleavage; TAD is the transactivation domain; SET is histone methyltransferase domain.

**Figure 2:**
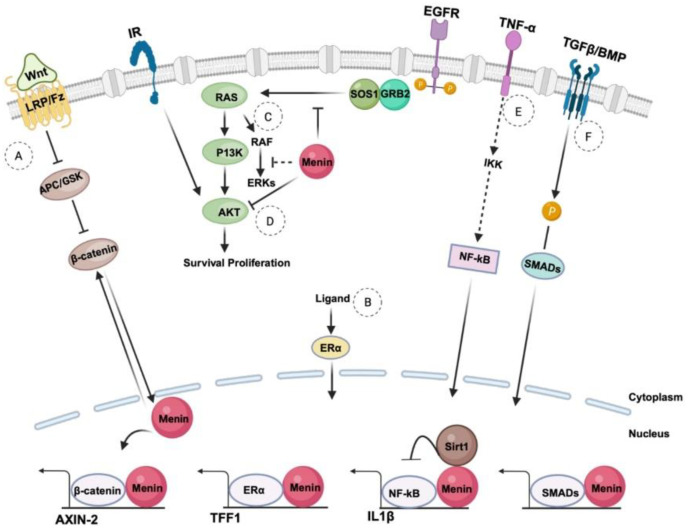
Menin and signaling pathways. (A) Menin participates in Wnt signaling by interacting with β-catenin which is located at the cell membrane and translocated to the nucleus in presence of Wnt. (B) Menin also participates in nuclear receptor signaling, directly interacting with ERα in a hormone-dependent manner and is recruited to the ERα target gene (TFF)1. It increases the active histone mark H3K4me3, recruiting MLL and activating target gene expression. (C) Menin inhibits extracellular signal-regulated kinase (ERK)-dependent phosphorylation, a downstream target in the Ras pathway. It inhibits receptor tyrosine kinase signaling in the cytoplasm by inhibiting AKT, SOS1-dependent activation of Ras, and suppression of extracellular signal-regulated kinase (ERK) activation. (D) In Akt signaling, menin suppresses both Akt1-dependent proliferation and antiapoptotic activity by reducing the translocation of Akt1 from the cytoplasm to the plasma membrane. (E) Menin interacts with nuclear factor (NF)kB and recruits Sirtuin (Sirt1) to deacetylate p65b and suppress NFkB-induced gene expression. (F) Menin regulates BMP signaling and TGF-β/SMAD pathways by interacting with SMAD3 or SMAD1/5 as a transcriptional co-regulator, enhancing or modulating the transcriptional activity of SMADs in response to the signaling cues from either TGF-β or BMP ligands. CDKN2B (p15) and CDKN1B (p27); TGF-β target genes; JUNB; and TGFB1 are some of the genes regulated by the MEN1-SMADs interaction. EGFR, epidermal growth factor receptor; GRB, growth factor receptor-bound; GSK3b, glycogen synthase kinase; ERα, Estrogen Receptor; SOS, son of Sevenless; RAF, rapidly accelerated fibrosarcoma; IKK, inhibitor of nuclear factor k-B kinase; BMP, Bone Morphogenic Protein; IL, interleukin; PI3K, phosphoinositide 3-kinase; TFF, trefoil factor; TNF, tumor necrosis factor. Bold arrows show strong activation or direct stimulatory action, whereas dotted arrows show indirect effect or tentative stimulatory action.

Three MLL regions have been experimentally shown to be crucial for leukemic transformation: the N-terminal region which binds to both menin (conserved across all MLL-fusion proteins) and lens epithelium-derived growth factor (LEDGF), the cystine-rich CXXC domain which mediates binding to non-methylated CpG DNA, and the polymerase-associated factor complex (PAFc) [6]. Menin specifically interacts with two fragments of MLL, the high-affinity motif MBM1 (menin-binding motif 1) and low-affinity MBM2 (menin-binding motif 2), located within an unstructured region at the N-terminus of MLL [7]. Both motifs are separated by a 7-glycine linker and likely bind to adjacent sites on menin, forming a bivalent interaction necessary for the leukemia-inducing activity of MLL fusion proteins. Disrupting this menin–MLL binding with small-molecule menin inhibitors (MIs) has evolved recently as a novel therapeutic strategy in treating AML [8]. The *MEN1* gene located on chromosome 11q13 is composed of nine introns and ten exons encoding the menin protein, which is 610 amino acids long, formed from an alternate splicing event after exon 2 (Figure 1A). Genetic and biochemical studies support a widely distinct expression profile and a tissue-specific function of *MEN1*. A large French collaborative study screened MEN1 syndrome and described 370 different variants from 1676 MEN1 patients [9]. Although no mutational hotspot was identified, some recurrent mutations were observed. Microdeletions, insertions, and indels resulting in frameshift consequences were the most common (42% of cases), followed by missense variants (26%) and nonsense variants (15%). Splice-site variants were the least reported. Chromatin immunoprecipitation studies reveal various human gene promoters of menin and identify *MEN1* as a target gene in different cancer cells, demonstrating the oncogenic functions during cancer progression [10,11]. Immunoprecipitation and next-generation sequencing (ChIP seq) confirm its occupancy at active transcription sites, which correlates with high gene expression. ChIP seq shows that menin directly interacts with promoters of different genes and influences the methylation of H3K4 associated with the promoters [12]. Menin is involved in binding to gene promoters, transcription factors, or enhancers at active transcription sites (Figure 1B). It links transcription factors to histone-modification pathways and functions as a global transcription regulator. Although it lacks intrinsic enzymatic activity, menin acts as a scaffold for various protein partners, influencing epigenetic gene regulation, modulating multiple regulatory pathways (Figure 2), and altering chromatin structure through histone modification [6].

Through the MLL complex, menin influences the expression of genes related to hematopoiesis and cell proliferation, such as *HOX* genes [13]. Menin binds to *JunD*, a component of the AP-1 transcription factor complex. This interaction typically represses *JunD*’s transcriptional activity, which promotes cell growth. The repression of *JunD* activity by menin is critical for its tumor-suppressive functions [14]. Menin forms complexes with SMAD proteins, specifically SMAD3, a key signaling mediator in the TGF-β (Transforming Growth Factor Beta) pathway. By interacting with SMADs, it modulates the transcription of genes involved in cell growth inhibition and apoptosis [15]. Menin can influence the activity of the NF-κB pathway, which is involved in immune responses and inflammation. This is important for regulating processes like cell survival and immune cell function. Menin also interacts with beta–catenin, a component of the Wnt signaling pathway, which is crucial for regulating cell proliferation, differentiation, and stem cell maintenance [16]. DOT1L is recruited to MLL fusion targets by menin, where it methylates H3K79, activating key oncogenes such as *HOXA9* and *MEIS1*. By regulating H3K79 methylation, this interaction influences chromatin accessibility and gene expression patterns essential for leukemogenesis and maintenance [17]. Menin also interacts with MYC, a transcription factor that regulates a plethora of genes involved in cell growth, proliferation, and metabolism. Menin can modulate MYC’s transcriptional activity. It has been shown to be a cofactor that enhances MYC-mediated transcription of target genes that promote cell proliferation and survival. Menin has been shown to interact with XPO1, a nuclear export receptor that may influence the nuclear export of various proteins involved in cell signaling and transcription. MLL fusion proteins can affect normal cellular processes, and the interaction between menin and XPO1 may play a role in its dysregulation. The nuclear export of transcription factors regulated by menin could lead to altered gene expression profiles that promote the proliferation and survival of leukemic cells [18].

Pediatric, adult, and therapy-related leukemias are associated with the chromosomal rearrangement at 11q23 that linked the implication of the lysine methyltransferase 2A (*KMT2A*) gene as a driver in AML [19]. *MLL*/*KMT2A* rearrangement constitutes more than 70% of infant acute lymphoblastic leukemia (ALL), ~35–50% of infant acute myeloid leukemia (AML), and 5–10% of adult AML cases. In about 10% of AML, partial tandem duplication (PTD) of exons encoding the N-terminus of *MLL* causes overexpression of MLL. *MLL* gain with non-*MLLr* is also occasionally seen in AML and myelodysplastic neoplasm [20]. Menin interacts with *MLL1* and *MLL2*; both harbor an SET domain with histone 3 lysine 4 (*H3K4*) methyltransferase activity. MLL can fuse with one of over 80 different fusion partners during this chromosomal translocation to form chimeric MLL fusion proteins proleukemogenic in *MLL*r leukemia. The most frequent *MLL* rearrangements involve *AF1P*, *AF17*, *AF9*, *AF4*, *ENL*, *AF6*, *SEPT6*, and *ELL* [21,22]. Menin’s direct interaction with the N-terminus of the MLL further recruits the fusion protein complex to activate leukemogenic *HOXA9* and its cofactor *MEIS1*, leading to oncogenic transformation. Disrupting this interaction between menin and *MLL*-FP has shown robust preclinical activity in multiple studies in *MLL*/*KMT2Ar* leukemia models. Interestingly, menin inhibition in WT-*MLL* AML, as with *NPM1*-m leukemia, has also been effective in early-phase studies. Conceivably, the downstream signatures like *HOXA9* and *MEIS1* are decreased in expression in both molecular subtypes of AML.

Clinically potent menin inhibitors (MIs) have completed first-in-human trials, opening new therapeutic opportunities. These novel agents tested in *KMT2A*r and *NPM1*-m AML have demonstrated meaningful clinical activities with approximately 30% response rates in the relapsed/refractory (R/R) setting and a considerable number showing complete response (CR) with no measurable residual disease (MRD). The safety profile of these inhibitors appears manageable. These evolving MIs are a significant breakthrough in the treatment of AML with promising activity and beg for an in-depth review of the evolution of this novel target, its implications in cellular homeostasis and tumorigenesis, development of targeted therapeutics, emergent resistance mechanisms to these novel drugs, newer approaches in the pipeline, and possible therapeutic combinations that are being explored.

### 1.2. Menin and Its Interacting Partners

Menin is retained within the nucleus via nuclear localization sequences (NLS1, NLS2, and NLS3) located near its C-terminus [22]. Typical NLSs consist of multiple positively charged amino-acid residues, and these basic residues specifically bind to a soluble transport receptor complex comprising importin *α* and importin *β*, leading to translocation into the nucleus. It has been shown that menin binds to DNA in a sequence-independent manner specifically using either C-terminus or other regions [22]. As menin associates with chromatin and the nuclear matrix, directly binding double-stranded DNA (dsDNA) and regulating gene expression, its nuclear localization should be essential for its role in the regulation of gene transcription [1].

Menin structure and interaction can reveal its tumor-suppressive function and context-dependent cellular functions. Interestingly, some of these interactions are, in fact, pro-proliferative in specific tissues, and hence, it is essential to study its interactive partner proteins. The key interaction partners of menin are shown visually in Figure 1B; they are divided into four classes and introduced below:

1. Transcription regulation: SMAD3, JunD, NF-kB, and MYC. Menin enhances the ability of SMAD3 to mediate TGF-β signaling, which is important for regulating cellular proliferation and differentiation, thereby contributing to tumor suppression [23]. Menin–SMAD3 complexes regulate the expression of several genes involved in cell cycle control and differentiation, including cyclin-dependent kinase inhibitors (CDKN1A) and p21 [23]. Additionally, this complex can also influence the expression of *HOX* genes, especially in the context of menin–MLL interactions. Menin also represses JunD at AP-1 sites, preventing excessive cell proliferation, and disruptions in this interaction can contribute to tumorigenesis. Additionally, menin suppresses NF-κB, a transcription factor involved in inflammatory responses, helping to regulate immune and pro-survival genes. Lastly, menin interaction with MYC and MLL fusion proteins drives the expression of oncogenic genes.
2. Epigenetic modifiers and chromatin regulators: SIN3A, HDAC1, SIRT1, and PRMT5. Menin functions as a nuclear scaffold and interacts with various chromatin regulators, including SIN3A, HDAC1, and PRMT5, to modulate histone modifications [24]. These interactions regulate gene silencing or activation, which is critical for maintaining cellular homeostasis. The menin–SIN3A–HDAC1 complex, for example, represses tumor suppressor genes through histone deacetylation.
3. DNA damage response and replication: RPA2 and FANCD2. Menin interacts with proteins involved in DNA repair, such as RPA2 and FANCD2, supporting its role in maintaining genomic stability. These interactions help ensure proper DNA replication and repair, and their disruption can contribute to the development of leukemia through genomic instability.
4. Signaling pathway modulators: Beta–catenin. Menin interacts with beta–catenin in the Wnt signaling pathway, and by fine-tuning this pathway, it regulates cell fate and proliferation [25].

#### 1.2.1. KMT2A

The menin central region residues in either KMT2A or the menin pocket are essential for menin–KMT2A interaction and *HOX* gene upregulation. It regulates *HOX* gene expression and broadly modulates the transcription of different genes associated with various genomic elements occupied by RNA polymerase II. [5]. KMT2A is a protein essential for normal development in hematopoietic cells. Its cleavage produces two subunits: the N- and C-terminus portions of KMT2A^N^ and KMT2A^C^ [26]. The C-terminal SET domain has the histone H3 lysine 4 (H3K4) methyltransferase activity and carries the NLS (Figure 1C). KMT2A binds to two and regulates transcription through histone H3K4 methylation. The MLL1 complex targets specific gene promoters and enhancers by interacting with other transcriptional machinery, leading to changes in chromatin structure. The *KMT2A* interaction with menin influences its binding to DNA, and both *KMT2A* and menin cause aberrant expression of homeobox genes, *HOX*, and their DNA-binding cofactor *MEIS1* [27,28]. *KMT2D* is another paralog of *KMT2A* that binds to menin and regulates distinct pathways as a tumor suppressor. It also activates the nuclear hormone receptors and modulates the transcription of the retinoic acid-responsive genes [29].

#### 1.2.2. LEDGF

Another essential protein for *MLL*-*AF9*-induced leukemia is LEDGF. The crystal structure of the menin–MLL1–LEDGF complex demonstrates that MLL1 is directly in contact with the menin N-terminus by forming a helix [30]. The menin–LEDGF complex plays a role in the recruitment of LEDGF to specific genomic loci, where it functions as a cofactor in transcriptional regulation.

#### 1.2.3. DOT1L

Menin interacts with the DOT1L (disruptor of telomeric silencing 1-like) protein to regulate histone methylation at lysine 79 on histone H3 (*H3K79*) residues. To maintain transcriptional activity, DOT1L, a histone *H3K79* methyltransferase capable of mono-, di-, and trimethylation, may counteract repressive histone deacetylases like SIRT1 [31]. Following MLL-ENL expression or mutation, the DOT1L link is supported by modulating H3K79me2 levels at MLL target genes. Menin can recruit DOT1L to target genes to activate transcription [32]. Dysregulation of the menin–DOT1L interaction has been implicated in leukemogenesis, particularly in *MLL*r leukemia. MLL fusion proteins recruit the menin–DOT1L complex to target genes, resulting in aberrant histone methylation and transcriptional activation of oncogenes involved in the leukemogenesis.

#### 1.2.4. RUNX1

The proleukemogenic genes *HOXA9* and *MEIS1* are also regulated by *RUNX1*. Menin can enhance the transcriptional activity of *RUNX1* by activating *RUNX1* target genes involved in leukemogenesis [33]. Dysregulation of the menin–*RUNX1* interaction has been linked to the pathogenesis of AML and ALL. Aberrant expression or function of menin or *RUNX1* can disrupt normal hematopoiesis and contribute to leukemic transformation [34].

#### 1.2.5. XPO1

XPO1 (Exportin 1), also known as CRM1 (Chromosome Region Maintenance 1), regulates the nucleocytoplasmic shuttling of several cargo proteins, including menin. This shuttling process is navigated by specific primary residue-rich amino acid sequences in the target proteins, an NLS, and a leucine-rich nuclear export signal (NES). The interaction between menin and XPO1 regulates the subcellular localization of menin. XPO1 inhibition can accumulate menin in the nucleus, altering its functions and affecting cellular processes. The selective inhibitors of nuclear export (SINE) covalently bind to cysteine 528 in the binding pocket of XPO1 and disrupt its nuclear export, leading to the accumulation of cargo proteins in the nucleus.

#### 1.2.6. MYC

**Figure 3:**
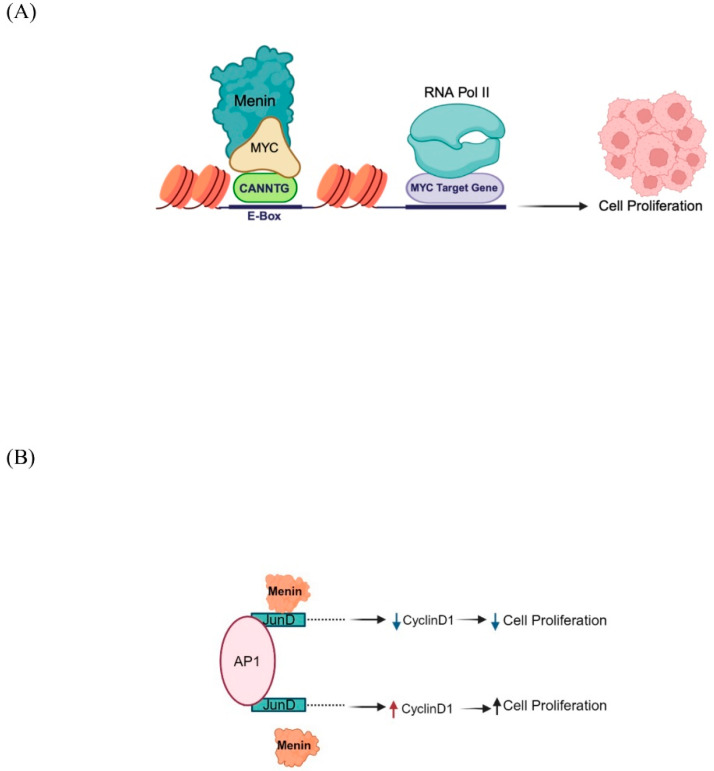
Menin and its interacting partners: (**A**) Menin enhances MYC-mediated transcription. It facilitates RNA Pol II phosphorylation to enhance MYC-mediated transcription and promote MYC-mediated cell proliferation. (**B**) Transcription factor JunD interacts with activated protein 1 (AP-1) and forms an active transcription complex. Interaction of menin with JunD downregulates cyclinD1, reducing cell proliferation.

MYC is a proto-oncogenic transcription factor that binds to the canonical E-box regions of almost 15% of human genes and is involved in their transcriptional regulation [35]. Menin’s interaction with *MYC* plays a crucial role in augmenting transcription, since menin binds directly to the E-boxes and engages with the TAD domain of *MYC*, thereby augmenting the expression of the *MYC* target gene (Figure 3). Menin stimulates cell proliferation by transcriptionally promoting the *MYC* target gene expression in cancer cells [36]. In this context, menin is pro-proliferative.

#### 1.2.7. JunD

Menin binds with JunD, a component of the AP1 transcription factor complex, and functions as a growth suppressor by impeding cell proliferation through the modulation of cyclin D1 promoter (Figure 3). In the absence of this interaction, *JunD* toggles the switch to being a growth promoter [37] rather than a suppressor. A histone deacetylase activity might also be implicated in repressing JunD-activated transcription. Several missense mutations in *MEN1* disrupt the menin–JunD interaction, suggesting a correlation between menin tumor suppressor function and its interaction with JunD to block JNK kinase-mediated JunD phosphorylation, which is critical for *JunD* activation [38]. JunD–menin interaction may also interfere with Ras-dependent cell transformation and oncogenesis [39].

In addition to these, menin interacts with double-stranded DNA sequentially using NLS1 and NLS2 regions [40]. Loss of these short sequences results in cell proliferation as menin fails to block the G2-M or G1-S phase. Menin also interacts with the S-phase kinase (ASK), which induces cell proliferation that appears to be contingent to the presence of menin. Menin is also associated with chromatin and the nuclear matrix. Increased accumulation of menin in the nuclear matrix in response to γ-irradiation substantiates its role in repairing DNA damage [41].

## 2. Developmental Therapeutics in HOX/MEIS1 Dysregulated AML

### 2.1. KMT2Ar and Menin Inhibition

*KMT2A*r leukemias can be phenotypically myeloid, lymphoid, or mixed lineage and are generally associated with poor prognosis. ALL with *KMT2A*r co-expresses myeloid markers, with negative expression of CD20 and deficient expression of CD22 corresponding to early stages of B-cell differentiation [42].

*KMT2A*r leukemias treated with standard therapies have a higher relapse rate and poorer outcomes [43]. Hematopoietic stem cell transplantation (HSCT) in eligible patients can salvage remissions, but the cure rate is still dismal. Likewise, infant *KMT2A*r leukemias are resistant to prednisone but sensitive to cytarabine [44]. Pediatric ALL treated with chemotherapy has a 5-year 90% overall survival (OS), whereas outcomes of *KMT2A*r infant leukemias remain vastly inferior at 20–44% [45] and further dismal in the R/R setting. Similarly, for adult *KMT2A*r ALL, the five-year OS rate is approximately 25%, with marginally improved outcomes following allogeneic stem cell transplant [46]. For AML with *KMT2A*r in adults, the OS rate with t(9;11) translocation ranges from 27% to 35%, while it is approximately 10% for all other translocations [47].

Genetic ablation of menin reverses aberrant *hox* gene expression in mouse *KMT2A*r leukemia models, which abrogates the differentiation arrest and oncogenic properties of *KMT2A*r. All KMT2A fusion proteins retain the menin binding site, which is essential for binding to *HOX* gene promoters. The inhibition of menin prevents the maintenance of high *HOX* gene expression and can revert the aberrant epigenetic state imposed by KMT2A fusion proteins. This reversion involves reducing the transcriptional activation of *HOX* genes, bringing their expression levels closer to those seen in normal hematopoietic cells. By restoring more normal *HOX* gene expression patterns, menin inhibitors can reverse the leukemogenic program driven by *KMT2A*r. This demonstrates the necessity of menin to maintain leukemogenesis but not normal hematopoiesis [48].

### 2.2. NPM1-m AML and Menin Inhibition

**Figure 4:**
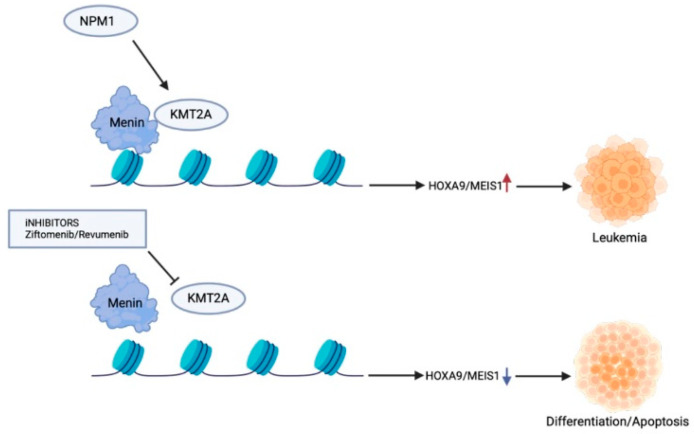
Mechanism of action of small-molecule menin inhibitors in *KMT2A*r AML and *NPM1*-m AML. Menin binds with KMT2A and overexpresses the *HOX* gene along with their cofactor *MEIS1*. *NPM1*-m turns exclusively cytoplasmic when mutated (NPM1c) with upregulation of *HOX* genes. Ziftomenib and revumenib (menin inhibitors) disrupt the menin–KMT2A interaction, suppressing *HOX* gene expression and inhibiting aberrant leukemogenesis.

*NPM1* mutations are frequent (20–30%) driver events in AML. These mutations are four base-pair frameshift insertions or duplications in exon 12 that lead to protein truncation and disruption of nuclear retention of *NPM1*. The OS rate of *NPM1*-m AML patients is about 40%, with a complete remission rate of approximately 80%, but 50% of patients will eventually relapse [18,49]. Fms-like receptor tyrosine kinase-3 internal tandem duplication (*FLT3*-ITD), which co-exists with *NPM1*-m, is the cause of poor survival and high relapse rates. *NPM1*-m AML can be treated using MRD-directed standard chemotherapeutic approaches with good outcomes [50], but co-occurring mutations significantly alter the prognoses. Different treatment options, disease biology, and age-related factors disappoint OS and disease-free survival in older *NPM1*-m patients than younger *NPM1*-m patients [51]. An increase in diversity of the immunoglobulin and T-cell receptor loci in homeostasis due to dysfunction of the lymphoid enzyme terminal deoxynucleotidyl transferase (TdT) is linked to *NPM1* and *FLT3* mutations [52]. *NPM1*-m is also associated with the upregulation of *HOXA* and *MEIS1* [53]. This altered leukemogenic program common between *NPM1*c and *KMT2A*r led to the hypothesis that menin is possibly associated with dysregulated expression of *HOX* and *MEIS* in *NPM1*-m AML, and targeting menin could be a therapeutic strategy (Figure 4). Gene editing studies have shown that *NPM1* mutant leukemias are dependent on menin and *MEIS1* for their leukemogenic function, and knocking out these genes disrupts the menin-*HOX-MEIS1* axis that leads to differentiation, loss of proliferation, and impaired leukemogenesis [54,55,56,57,58].

### 2.3. Other HOX Dysregulated Signatures and Menin Inhibition

Leukemias characterized by mutations in *EZH2*, *DNMT3A*, or *ASXL1* or *SET*-*NUP214* or *RUNX1*-*EVI1* fusions also show overexpression of *HOXA* and *MEIS1* [59,60,61,62], and it might hypothetically be reasonable to expect a response to MI; future clinical studies will provide more confirmation about these novel targets. In support of this, investigators recently showed that *NUP98*-rearranged leukemias responded to potent menin inhibition in preclinical mouse and PDX models [63]. In AML, *HOX* is influenced by recurrent chromosomal translocations involving direct fusion of *HOX* with nucleoporin genes such as *Nucleoporin 98* (*NUP98*) and upstream regulators, like MLL-NUP98 fusion oncoproteins [64]. Chromosomal translocations cause oncogenic NUP98 gene fusions, and these fusions involve intrinsically disordered and N-terminal regions of *NUP98* with over 30 partner genes. One of the most well-studied is *NUP98*-*HOXA9* from the translocation t(7;11)(p15;p15). Changes in chromatin structure and gene expression mediate leukemogenesis driven by NUP98 [65]. Preclinical studies show that *NUP98*r leukemia is sensitive to menin inhibitors in vivo [63]. A few other *HOX*/*MEIS1* dysregulated signatures are listed in Table 1.

## 3. Menin Inhibitors in Clinical Trials

Several MI trials evaluate monotherapy’s safety, tolerability, and efficacy or combined it with other standard-of-care treatments in AML primarily and beyond (Table 2). Many of these clinical trials have limited enrollment in expanding cohorts beyond *KMT2A*r and *NPM1*-bearing mutations. However, some ongoing investigator-initiated clinical trials include other signatures. MI has the potential to reach a more significant subset of acute leukemia with similar dependency as the menin–KMT2A interaction thanks to the improved precision approaches testing characteristic gene expression. The first-in-human phase I/II clinical trials began in 2019, evaluating different novel MI: KO-539, SNDX-5613, JNJ-75276617, and DS-1594b, some of which will be highlighted here.

## 4. Monotherapies with Menin Inhibition

### 4.1. Ziftomenib

Ziftomenib (KO-539) is an effective, potent small-molecule inhibitor of the menin–KMT2A binding site. A phase I trial (KOMET001 (NCT04067336) continues recruiting patients with R/R AML. The earlier dose escalation phase Ia (*KMT2A*r, *NPM1*-m, and other signatures included), phase Ib dose validation (*KMT2A*r or *NPM1*-m), and phase Ib dose expansion cohort (*NPM1*-m only) included heavily pretreated AML patients, and 25% of them had prior HSCT. At the data cutoff in October 2022, the phase Ib dose validation portion of the study (testing 200 mg and 600 mg) reported a 30% CR rate in the *NPM1*-m cohort that received 600 mg of ziftomenib, while there was only 5% CR/CRh (n = 18) in the *KMT2A*r cohort. Despite the on-target differentiation syndrome, including a grade 5 AE in the *KMT2A*r arm, it showed a favorable safety profile and tolerability at the 600 mg dose level. There were no grade 3 or higher treatment-emergent adverse effects in patients harboring *NPM1*-m at either dose level. The updated results from the phase Ib dose expansion cohort with *NPM1*-m patients (n = 20) at the data cutoff of April 2023 also demonstrated consistent responses, with an ORR of 45% and a CR rate of 35%.

With the RP2D of 600 mg, the trial is now recruiting the phase II portion, with only *NPM1*-m patients. To note, the drug is currently studied in combination with other AML therapies in *KMT2A*r AML, whereas its development is currently more geared towards *NPM1*-m leukemia as monotherapy.

### 4.2. Revumenib

Revumenib (SNDX-5613) is another oral menin–KMT2A inhibitor and analog of VTP-50469 that exhibits potent preclinical antileukemic activity in *KMT2A*r and *NPM1*-m AML, including long-term remissions after treatment termination [55]. The safety and efficacy of revumenib was evaluated in the AUGMENT-101 phase I/II study for R/R *KMT2A*r or *NPM1*-m AML and ALL. The dose escalation part in the phase 1 study (N = 68) (NCT04065399) included heavily pretreated patients (four median lines of prior therapy), and almost half of them (46%) had received prior allo-HSCT [76]. Most of the patients had AML (82%), and a few had ALL (16%) and MPAL (2%). The study had a majority of *KMT2A*r (68%) patients compared to *NPM1*-m (21%) or wild type for both (12%). Since revumenib is a CYP3A4 substrate, two parallel dose-escalation cohorts were conducted with and without a potent CYP3A4 inhibitor. The only dose-limiting toxicity observed in both arms was grade 3 QT interval prolongation (over 500 mg) without any clinical symptoms. Febrile neutropenia (31%), sepsis (18%), and thrombocytopenia (19%) were the most frequent grade 3 or higher treatment-emergent adverse events (TEAEs). The differentiation syndrome was seen only in 16% of patients. The overall response rate and CR/CRh was 53% and 30% (*KMT2A*r 59% and 33%; *NPM1*-m 36% and 21%, respectively) in the entire cohort.

### 4.3. JNJ-75276617

Another potent, oral, and selective inhibitor, JNJ-75276617, is also being studied in a first-in-human ongoing trial to establish the RP2D (NCT04811560) [77]. As of April 2023, fifty-eight patients have been treated with the drug, fifty-six (97%) with R/R AML and two (3%) ALL, with *NPM1*-m in twenty-five (43%) patients and *KMT2A*r present in thirty-three (57%). Five (9%) patients had DLTs, while two (3%) had differentiation syndrome. Reduction in bone marrow disease burden was reported in twenty-six of forty-one patients (63%), and over one-third showed a >50% decrease in BM blasts. The ORR (≥PR) was 50% (n = 4), with all the ongoing responses reported at the highest dose level with ≥3 pts (90 mg BID; n = 8). There were twelve responders in all cohorts, including one MRD-negative CR. Eight responders continued with the treatment, while one patient discontinued for allogeneic SCT. Downregulated expression of menin–KMT2A target genes and upregulation of associated differentiation genes (ITGAM, MNDA) was evident in responding patients. With treatment, *KMT2A*-r cells or *NPM1* variant allele frequency (VAF) was reduced compared to baseline. Somatic mutations in *MEN1,* such as M327I and T349M, have been observed in patients with acquired clinical resistance to MI revumenib. JNJ-75276617 can still displace KMT2A and prevent its interaction between KMT2A and wild-type menin or menin bearing the M327I or T349 mutation [78]. This seems to be a distinctive feature of JNJ-75276617, though further rigorous clinical studies are required to confirm its efficacy in resistant cases.

### 4.4. DSP-5336

DSP-5336 is a small molecule currently under phase I/II clinical development (DSP-5336-101 trial) enrolling adult patients with R/R AML or ALL and dose-expansion cohorts enrolling patients with R/R AML with *KMT2A*r or *NPM1*-m. Based on the data cutoff date in May 2024, an overall ORR was reported at 57% and a CR rate of 24%. More specifically, patients with *NPM1*-m (n = 9) achieved a 44% ORR and a 33% CR rate, while patients with *KMT2A*r R/R AML who received at least 140 mg of the study drug (n = 12) experienced an ORR of 67% and a 42% composite CR rate. There were no reported dose-limiting toxicities, treatment-associated discontinuations, treatment-associated QT prolongation, or cardiac side effects. Additionally, DSP-5336 did not report any clinically significant differentiation syndrome (n = 3).

### 4.5. BMF-219

BMF-219, a covalent binding menin inhibitor, is being tested in adult AML and ALL patients with *KMT2A*r or *NPM1*-m, diffuse large B-cell lymphoma (DLBCL), chronic lymphocytic lymphoma (CLL), and multiple myeloma (MM) (COVALENT-101, NCT05153330). By July 2023, a total of twenty-four AML and two ALL patients with a median of four prior lines of therapy had been enrolled in the trial; eleven patients had SCT in the past. BMF-219 was well tolerated; no dose-related toxicities or dose-associated discontinuations were noted. Differentiation syndrome was observed in 13% of the cases. Another phase I/II trial (NCT06052813) using BMF-219 recruiting patients R/R *KMT2A*r or *NPM1*-m AML and MPAL/ALL with *KMT2A*r is estimated to be completed by 2027.

## 5. Combination Studies

Treatment of acute myeloid leukemias using combination therapies has historically shown better results by building synergies. Combining selumetinib (targeting MEK1/2) with VTP-50469 is synergistic in preclinical models harboring *KMT2A*r and *RAS* mutations [79].

Many investigator-initiated trials are currently exploring various combinations with menin inhibition. Ziftomenib (KOMET-007/ NCT05735184) is being investigated to assess its safety, tolerability, and preliminary antileukemic activity in combination with venetoclax and azacitidine (ven/aza), ven, and 7 + 3 for two different molecularly defined AML subgroups: *NPM1*-m and *KMT2A*r in the relapsed/refractory setting. KOMET-008 (NCT06001788) is another ongoing dose escalation and expansion study to check the tolerability, safety, and preliminary efficacy of ziftomenib with FLAG–idarubicin or low-dose cytarabine or gilteritinib for the treatment of either *NPM1*-m or *KMT2A*r or *NPM1*-m with *FLT3*^MT^ R/R AML.

Combining menin inhibitors with different inhibitors, such as CDK6, CDK9, XPO1, BET, MOZ, LSD1, and CBP/p300, also induces synergistic lethality in AML cells harboring different mutations [80,81]. Many of these co-dependencies are also transcriptional targets for *MEIS1*, such as *FLT3*; hence, their combination is possibly synergistic. They also seem to abrogate drug resistance, leading to longer remission [82].

Revumenib with fludarabine and cytarabine chemotherapy is currently recruiting patients (AUGMENT-102 trial, NCT05326516). In an ongoing phase I/II trial SAVE study (NCT05360160) designed for *KMT2A*r, *NPM1*-m, and *NUP98*r leukemias, MRD was reported to be undetectable in three of seven treated patients (43%) with the combination. Out of three responding patients who underwent SCT, two are still in remission, one is continuing maintenance, and one died due to complications from the SCT before starting maintenance. Grade 3 adverse events were thrombocytopenia (25%), febrile neutropenia (63%), and neutropenia (25%). Two patients developed a grade 2 differentiation syndrome, which resolved with steroids [83]. In newly diagnosed patients, a phase II study using revumenib, daunorubicin, and cytarabine is recruiting patients (NCT05886049). A phase I study is investigating the addition of revumenib to the standard induction of 7 + 3 + Midostaurin (NCT06313437) in the frontline AML treatment setting. Similarly, in the R/R setting, a phase I trial is investigating the use of revumenib and gilteritinib (NCT06222580). In pediatric AML populations, three phase I trials are investigating the use of menin inhibitors: ziftomenib with venetoclax and gemtuzumab (NCT06448013), ziftomenib with fludarabine and cytarabine (NCT06376162), and revumenib in conjunction with chemotherapy (fludarabine, cytarabine, IT methotrexate, or cytarabine and asparaginase, NCT05761171).

Preclinical studies from our group combining XPO1 inhibitor (selinexor) and menin inhibitor (ziftomenib) inhibited *MLL*-r and *NPM1*-m AML cells in vitro and in vivo [81]. Such preclinical results set a rationale to translate the use of the drug combinations in the R/R AML setting.

## 6. Menin Resistance

Like any targeted agents, resistance to menin inhibition is not uncommon. Resistance may arise due to mutations in the binding sites of menin, which prevents effective interaction between the inhibitor and its target. The menin inhibitor interaction affinity is decreased due to these mutations preventing drug-induced displacement of the menin–MLL1 complex from chromatin, abrogating gene expression changes [84]. Acquired point mutations in *MEN1* residues, including M327, T349, G331, and S160, have been described to impair the menin–MLL interaction, thereby leading to resistance [84]. Alternatively, using a CRISPR knockout library, a second resistance mechanism that involves deletions in polycomb repressive complex 1 (PRC1.1), bypassing the need for menin–MLL1 inhibition and aberrantly activating the *MYC* oncogene has been also described [85]. However, alternative resistance patterns have also been noticed in the absence of these mutations [86]. The AUGMENT-101 trial showed resistance to menin inhibition in the absence of somatic *MEN1* mutations, suggesting other mechanisms of resistance [76]. Emerging evidence suggests that a form of nongenetic cellular adaptive resistance might be able to bypass the dependence on menin [50]. The details of this and other potential forms of resistance are yet to be investigated [87]. Crispr–Cas9 studies inducing similar mutations showed resistance to menin inhibitors in the MOLM-13 cell line [80]. Some patients can be primarily resistant, while some develop secondary resistance after a brief exposure to menin inhibition. Understanding and overcoming these are critical for improving treatment outcomes.

After an initial response to revumenib, the AUGMENT-101 clinical trial showed a relapsing disease with somatic mutations in the MEN1 gene. The study focused on detecting *MEN1* somatic mutations observed in several patients that were not detectable. *MEN1* mutations (M327, G331, T349, and S160) were observed in xenograft studies with prolonged drug exposure similar to the observation in patients [84]. In addition to its oncogenic activity in *KMT2A*r and *NPM1*-m leukemia, it can also directly interact with histones and read nucleosomal methylated *H3K79me*2 [88].

About 40% of resistant cases showed a mutation in *MEN1*, whereas it was unaltered in others, denoting the presence of other escape resistance mechanisms. Recurrent mutation in the *MEN1* gene has also been a reason for resistance towards revumenib. A phase I dose escalation clinical trial of menin inhibitor revumenib in 68 patients with R/R AML with *KMT2A*r or *NPM1*-m showed partial recovery of 30% of patients [87]. Menin mutations conferred resistance not only to revumenib but also to various structurally distinct menin inhibitors. One intriguing aspect is resistance associated with decreased expression of *HOX* and *MEIS* genes. These genes are typically upregulated in leukemias with *KMT2A*r, and their downregulation in the context of menin inhibition can impact resistance mechanisms.

## 7. Differentiation Syndrome

Differentiation syndrome (DS) is a potentially life-threatening complication more recognized since the advent of differentiating therapeutic agents such as ATRA (all trans retinoic acid) used in the treatment of acute promyelocytic leukemia (APL). It was subsequently observed more broadly with targeted therapeutics in AML. DS has been reported with menin inhibitors with both *NPM1* mutant and *KMT2A*r leukemias [87,89] with a similar presentation as in APL or even more pronounced [90,91,92].

Early identification and initiation of high-dose corticosteroids will be the cornerstone in the management of DS [93]. More specific clinical management of DS with these novel menin inhibitors will continue to evolve as more patients are being treated in clinical trials.

## 8. Conclusions and Future Directions

While menin inhibitors hold potential as targeted therapies for cancer, challenges remain, including identifying predictive biomarkers for patient selection, optimizing drug dosing and scheduling, and managing potential on- and off-target toxicities. Although the downregulation of *HOXA9* and *MEIS1* somewhat indicates a response to MI, some patients do not respond despite lowering the *HOX*/*MEIS* program expression. Ongoing research efforts continue to address these challenges and refine the clinical development of menin inhibitors. Numerous works in the target discoveries in AML have allowed these small-molecule inhibitors to reach clinical investigation. Proteolysis-targeting chimeras (PROTACs) to degrade MEN1 could be a prospective approach with potentially broader antitumor activity than specific menin–KMT2A inhibitors [94]. Furthermore, optimal combination strategies and their manageable toxicity could expand efficacy and encourage innovative therapeutic approaches. Many agents, either standard or investigational, could be ideal partners.

## Figures and Tables

**Table 1:** Genetic alterations with the *HOX/MEIS1* gene expressions and dysregulated signatures.

Mutations/Alterations	Upregulated Expression of Genes	Downregulated Expression of Genes
MLL fusion [66]	*HOXA7*, *HOXA9*, *HOXA10*, *HOXA11*, *MEIS1*	*HOXA1*, *HOXA2*, *HOXA4*, *HOXA6*
*PICALM*-*MLL10* [67]	*HOXA5*, *HOXA9*, *HOXA10*, *MEIS1*	*HOXB9*, *HOXC11*, *HOXC13*
*NPM1*-m [68]	*HOXA4*, *HOXA5*, *HOXA6*, *HOXA7*, *HOXA9*, *HOXA10*, *HOXB2*, *HOXB3*, *HOXB5*, *HOXB6*, *PBX3*, *MEIS1*	*CD34*
*NPM1*-m [69]	*HOXA1*, *HOXA2*, *HOXA3*, *HOXA4*, *HOXA5*, *HOXA7*, *HOXA9*, *HOXA10*, *HOXA11*, *HOXA13*, *HOXB2*, *HOXB3*, *HOXB4*, *HOXB5*, *HOXB6*, *HOXB8*, *HOXB9*, *HOXD3*, *HOXD13*, *MEIS1*, *PBX3*	*CD34*
*NPM1*-m [70]	*HOXA1*, *HOXA2*, *HOXA3*, *HOXA4*, *HOXA5*, *HOXA7*, *HOXA9*, *HOXA10*, *HOXA11*, *HOXB2*, *HOXB3*, *HOXB4*, *HOXB5*, *HOXB6*, *HOXB7*, *HOXB9*, *MEIS1*, *PBX3*	*CD34*
*NPM1*-m [71]	*HOXA2*, *HOXA5*, *HOXA6*, *HOXA7*, *HOXA10*, *HOXB2*, *HOXB3*, *HOXB6*, *MEIS1*, *PBX3*	*CD34*, *C4*
*PTPML*–*RARA* [72]	-	*HOXA1*, *HOXA4*, *HOXA5*, *HOXA7*, *HOXA11*, *HOXB3*, *HOXB7*, *HOXB8*, *HOXC6*, *HOXC9*, *HOXC11*, *HOXC12*, *HOXC13*, *HOXD4*, *HOXD8*, *HOXD13*
*NUP98*-*MLL* [73]	-	*HOXA5*, *HOXA7*, *HOXA9*, *HOXA10*, *MEIS1*
*SET*-*NUP214* [62]	*HOXA5*, *HOXA9*, *HOXA10*, *HOXA11*	
*BAALC* high expresser [74]	*CD34*	*HOXB5*, *HOXB6*, *HOXB7*, *HOXB9*
*CEBPA*mut [75]	*CD34*	*HOXA1*, *HOXA2*, *HOXA3*, *HOXA4*, *HOXA5*, *HOXA6*, *HOXA7*, *HOXA9*, *HOXA10*, *HOXB2*, *HOXB3*, *HOXB4*, *HOXB5*, *HOXB6*, *MEIS1*, *PBX3*

**Table 2:** Menin inhibitors currently available in phase I/II clinical trials in acute myeloid leukemia patients.

Identifier	Drugs	Trial Phase	Genetic Signatures	Status
NCT04067336	Ziftomenib	I/II	Menin-*MLL*(*KMT2A*)	Recruiting
NCT05735184	Ziftomenib + Venetoclax, Azacitidine	I	*NPM1*-m, *KMT2A*	Recruiting
NCT06001788	Ziftomenib	I	*NPM1*-m, *KMT2A*, *FLT3*	Recruiting
NCT06052813	BN104	I/II	*KMT2Ar*, *NPM1*-m	Recruiting
NCT05153330	BMF-219	I	*KMT2A*r, *NPM1*-m	Recruiting
NCT04067336	Ziftomenib	I/II	*NPM1*-m	Recruiting
NCT05360160	SNDX-5613 + ASTX727 + Venetoclax	Ib/II	*KMT2A*r or *NUP98*r, or *NPM1c*	Recruiting
NCT04811560	Bleximenib	I/II	*KMT2Ar*, *NPM1*-m, *NUP98* or *NUP214*	Recruiting
NCT06222580	SNDX-5613, Gilteritinib	I	*FLT3*	Recruiting
NCT05886049	SNDX-5613 + Daunorubicin + Cytarabine	Ib	NPM1-m*/FLT3-ITD/FLT3-TKD*	Recruiting
NCT05761171	Revumenib	II	*KMT2A*r	Recruiting
NCT06440135	Ziftomenib	I	*KMT2Ar*, *NPM1*-m	Not Yet Recruiting
NCT05738538	Ziftomenib	Expanded Access Program	*NPM1*-m	Not Yet Recruiting
NCT06313437	Revumenib, Midostaurin, Cytarabine, Daunorubicin	I	*NPM1*-m, *FLT3*	Not Yet Recruiting
